# *In Vivo* Evaluation of Cervical Stiffness Evolution during Induced Ripening Using Shear Wave Elastography, Histology and 2 Photon Excitation Microscopy: Insight from an Animal Model

**DOI:** 10.1371/journal.pone.0133377

**Published:** 2015-08-28

**Authors:** Laura Peralta, Eve Mourier, Christophe Richard, Gilles Charpigny, Thibaut Larcher, Dora Aït-Belkacem, Naveen K. Balla, Sophie Brasselet, Mickael Tanter, Marie Muller, Pascale Chavatte-Palmer

**Affiliations:** 1 Department of Structural Mechanics, University of Granada, Granada, Spain; 2 INRA, UMR 1198 Biologie du Développement et Reproduction, Jouy en Josas, France; 3 PremUp foundation, 75006 Paris, France; 4 INRA, UMR 703 APEX, Oniris, F-44307 Nantes, France; 5 Aix-Marseille Université, CNRS, Centrale Marseille, Institut Fresnel UMR 7249, 13013 Marseille, France; 6 Institut Langevin, ESPCI ParisTech, CNRS, Université Paris Diderot - Paris 7, Paris, France; 7 Department of Mechanical and Aerospace Engineering, North Carolina State University, Raleigh, North Carolina, United States of America; Tufts University, UNITED STATES

## Abstract

Prematurity affects 11% of the births and is the main cause of infant mortality. On the opposite case, the failure of induction of parturition in the case of delayed spontaneous birth is associated with fetal suffering. Both conditions are associated with precocious and/or delayed cervical ripening. Quantitative and objective information about the temporal evolution of the cervical ripening may provide a complementary method to identify cases at risk of preterm delivery and to assess the likelihood of successful induction of labour. In this study, the cervical stiffness was measured *in vivo* in pregnant sheep by using Shear Wave Elastography (SWE). This technique assesses the stiffness of tissue through the measurement of shear waves speed (SWS). In the present study, 9 pregnant ewes were used. Cervical ripening was induced at 127 days of pregnancy (term: 145 days) by dexamethasone injection in 5 animals, while 4 animals were used as control. Elastographic images of the cervix were obtained by two independent operators every 4 hours during 24 hours after injection to monitor the cervical maturation induced by the dexamethasone. Based on the measurements of SWS during vaginal ultrasound examination, the stiffness in the second ring of the cervix was quantified over a circular region of interest of 5 mm diameter. SWS was found to decrease significantly in the first 4–8 hours after dexamethasone compared to controls, which was associated with cervical ripening induced by dexamethasone (from 1.779 m/s ± 0.548 m/s, *p* < 0.0005, to 1.291 m/s ± 0.516 m/s, *p* < 0.000). Consequently a drop in the cervical elasticity was quantified too (from 9.5 kPa ± 0.9 kPa, *p* < 0.0005, to 5.0 kPa ± 0.8 kPa, *p* < 0.000). Moreover, SWE measurements were highly reproducible between both operators at all times. Cervical ripening induced by dexamethasone was confirmed by the significant increase in maternal plasma Prostaglandin E2 (PGE2), as evidenced by the assay of its metabolite PGEM. Histological analyses and two-photon excitation microscopy, combining both Second Harmonic Generation (SHG) and Two-photon Fluorescence microscopy (2PF) contrasts, were used to investigate, at the microscopic scale, the structure of cervical tissue. Results show that both collagen and 2PF-active fibrillar structures could be closely related to the mechanical properties of cervical tissue that are perceptible in elastography. In conclusion, SWE may be a valuable method to objectively quantify the cervical stiffness and as a complementary diagnostic tool for preterm birth and for labour induction success.

## Introduction

Despite numerous advances and intensive research in perinatal medicine, spontaneous preterm birth (PTB) is the leading global cause of neonatal mortality, and premature babies who survive are at risk of its morbidity consequences [1]. According to the World Health Organisation (November 2014), PTB refers to a labour that occurs at a gestational age of less than 37 completed weeks (< 259 days), and is defined by the association of uterine contractions and cervical modifications leading to the delivery of a preterm infant. Although spontaneous PTB is a heterogeneous syndrome, precocious cervical softening, shortening and dilatation are a common denominator.

On the other hand, labour has to be induced in approximately 23% of the pregnancies worldwide [2]. The induction of labour is recommended in circumstances in which the risks of waiting for the onset of spontaneous labour are judged by clinicians to be greater than the risks associated with shortening the duration of pregnancy by induction [3]. Induction, compared to expectant management, may substantially reduce the neonatal mortality [4]. Induction, however, fails in 32% of the induced pregnancies [2]. As a consequence, cesarean section is performed. Cesarean delivery not only carries operative risks, but also increases risks for future pregnancies [5].

Therefore, the control of cervical ripening has been considered for the last 30 years as one of the most pressing problems in obstetrics [6, 7]. So far, however, no reliable clinical tool is available for quantitative and objective evaluation of cervical softness. In current clinical practice, this biomechanical status is only assessed by digital palpation, using a subjective scoring system introduced by Bishop [8]. Nevertheless, this assessment is subjective and several studies have shown that the Bishop score is a poor predictor for induction success [9] and does not provide accurate diagnosis of imminent PTB [10]. In the past decade, some studies have reported that transvaginal sonographic measurement of cervical length appears to be a better predictor of PTB than the Bishop score, but this technique is a poor indicator of preterm labour [11, 12] and of the outcome of childbirth (cesarean delivery) [13, 14]. The measured geometrical changes yield a low percentage of accurate predictions. They provide a limited anticipation of the cervix ripening and are incompetent to assess the mechanical changes [7].

Quantitative information about the temporal evolution of the cervical ripening is practically nonexistent in the literature. Nevertheless, the use of tonometric techniques such as proposed by [15] and [16], and more recently [17, 18], using an aspiration device, demonstrated that cervical softening occurs in a gradual fashion throughout gestation.

Elastography has recently been used in obstetrics and gynaecology in a quest for meaningful information on the degree of cervical stiffness/softness, which in addition to cervical length, may provide a complementary method to identify cases at risk of preterm delivery and assess the likelihood of successful induction of labour. Quasi-static elastography is an imaging technique where displacements in the tissue are generated by hand using the probe. The displacement and the generated strain in the tissue are then estimated using two-dimensional correlation of ultrasound images, and the resulting strain is represented as a colour map. Lately, many authors have proposed quasi-static elastography as a new method to evaluate the softening of cervical tissue [19–22], but all studies concluded that quasi-static elastography has a limited interest for cervical assessment. Indeed, the resulting colour map is only a qualitative description of the relative strain distribution, far away from a quantitative description of the real stiffness of the tissue. Despite the simplicity and compatibility of the technique with standard equipment, measurements are dependent on transducer pressure applied by the operator and this technique would be more useful if measurements could be standardised between subjects. Therefore, so far, quasi-static elastography cannot reliably quantify cervical stiffness and cannot be used to predict PTB. Absolute values of cervical stiffness are necessary for an objective assessment of the cervical mechanical state, and the subsequent diagnosis of PTB or predictor for induction success [7, 23].

As opposed to static elastography, dynamic elastography is based on the propagation of shear waves within the tissue. Shear wave speed (SWS) estimation has minimal dependence on user interaction and enables the targeting of a specific area of evaluation [24]. In a homogeneous tissue, measuring the SWS propagation allows the quantification of stiffness. Various techniques have been developed using different modalities, employing different ways to generate shear waves and extracting different parameters of tissue motion. According to the terminology that is currently used in the literature [24], the term Shear Wave Elastography (SWE) only refers to methods that make images of shear wave speed using radiation force excitation, even though all dynamic methods use shear waves. The point shear wave elastography (pSWE) also uses acoustic radiation force to generate shear waves, but unlike SWE, the resulting value is only a point measure in a specific region of interest and not an image. Recently, *Carlson et al.* used pSWE in both, *ex vivo* and *in vivo* human cervix to assess softness/stiffness [25], pre- and post-labour induction [26]. They concluded that shear waves are sensitive enough to differentiate between ripened and unripened cervical tissue, and thus showing that shear waves might be a valuable diagnostic tool for the objective quantification of cervical stiffness/softness. In this study, we propose, for the first time, to evaluate the cervical stiffness evolution during labour induction in an animal model using SWE.

Understanding the mechanisms that take place in normal pregnancy will allow a better comprehension of the cervical remodelling and lead to better methods of diagnosis of PTB and successful induction of labour. The cervix undergoes important changes throughout the gestation process. Just before birth, the final remodelling of the cervix is driven through the secretion of prostaglandins by the fetoplacental unit. Prostaglandins (PGs) are derived from arachidonic acid and consist of two main families, i.e., PGE and PGF. Upon the increased secretion of cortisol by the fetal adrenals, prostaglandin synthase (PGHS)-2 gene expression in the placenta is up-regulated, resulting in increased production of PGs [27]. In particular, in the cervical region, there is an increased production of PGE2 and subsequent matrix remodelling [27]. Along with the decrease in elasticity, several changes occur in the structure of the tissue (both biochemically and morphologically). Previous studies have reported on the complex evolution of the collagen network in the cervix during the gestation [28, 29]. However, the link between stiffness and the cervical remodelling is not yet fully understood. To gain a better insight into molecular scale processes, optical microscopy has been recently applied [30]. Second Harmonic Generation (SHG) and Two-photon Fluorescence microscopy (2PF) are optical microscopy contrasts that are now widely used for label free imaging in tissues at the sub-micrometric scale. These two processes rely on the excitation of a tissue sample by a pulsed near infra red illumination, with considerable advantages in terms of penetration depth that can reach today up to a millimetre under pure optical excitation. Under two-photon excitation at wavelengths in the range [760nm–950nm], 2PF originates from cell proteins autofluorescence (e.g. amino acids like tryptophan and tyrosine, riboflavins, nicotinamides), cell metabolic activity, but also from collagen and elastin. 2PF has been used as a spectral ruler for tumorous tissues, in particular for monitoring collagen re-structuration during pregnancy [31, 32] or for the screening and diagnosis of cervical pre-cancer [33]. While 2PF does not require any particular molecular organisation to occur, SHG, in contrast, is only specific to non-centrosymmetric arrangements, with a particularly high efficiency and specificity in fibrillar type I collagen [34–36]. SHG microscopy has made possible sub-micrometric resolution imaging of complex molecular organisation over millimetric size regions of a tissue, relating for instance collagen morphology to cancer and pathology developments [37–41].

Fibrillar collagen types I and III are the main structural protein of the cervix, which undergo a large transformation changes over pregnancy and labour [42, 43]. Recent studies performed in the mice and human cervix have emphasised the intrinsic relation between the cervix stage and collagen organisation. In particular collagen fibre size progressively increases from early to late pregnancy, while at the micrometric scale, pores between collagen fibres become larger and farther apart [28]. A major advantage of two-photon microscopy is the possibility to combine both 2PF and SHG contrasts simultaneously, making multi-contrast label-free morphological imaging possible in complex molecular structures. While today, 2PF/SHG microscopy is widely exploited as a complementary technique to histological studies in the study of cancers developments and tissue diagnosis, there is however no report yet on the combination of both 2PF/SHG contrasts for the cervix optical imaging.

Having objectively defined time control over the cervical maturation may be difficult in humans. Animal models, in particular sheep, have been largely used for the exploration of mechanisms of cervical function and parturition [44]. Their size similar to humans enables the use of tools developed for humans, their physiology has been well explored and they enable accurate and easy control of the variables. In the present work, we propose an assessment of the evolution of the stiffness during the cervical maturation process using pregnant sheep as a model, employing SWE. Histological analysis and two-photon excitation microscopy, combining both SHG and 2PF contrasts, were used to report, at the microscopic scale, the integrity of cervical tissue. The main goal was, on the one hand to link structural changes with tissue elasticity, and on the other had to study the feasibility of SWE to objectively quantify the cervical stiffness during the maturation process and, ultimately, be a diagnostic tool for PTB and for labour induction success.

## Materials and Methods

### Animals

#### Ethics Statement

The experiment was performed in accordance with the International Guiding Principles for Biomedical Research involving Animals as promulgated by the Society for the Study of Reproduction and in accordance with the European Convention on Animal experimentation. The local ethical committee (name: Comethea, registered under ethical committee *N*°45 in the French national register) approved the experimental design, which was registered under N° 00264.01). All animals were unconscious prior to exsanguination.

The study was conducted on 9 pregnant ewes (Ile de France breed), maintained at the National Agricultural Research Institute (Institut National de la Recherche Agronomique, INRA), at 127 days of pregnancy (term = 145 days in sheep). Animals were randomly allocated to one of two groups: dexamethasone (n = 5) or control group (n = 4). Food and water were provided ad libitum throughout the experiment. Each ewe was permanently identified by an eartag.

**Fig 1:**
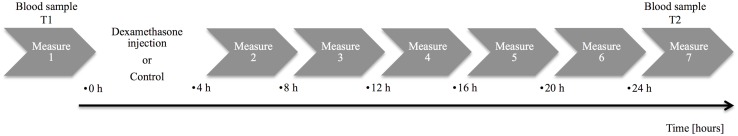
Schematic representation of the experimental set-up.

All animals were examined every 4 hours for 24 hours using ultrasound transvaginal examination (see Fig 1). The animal was removed from the pen where all ewes were kept, and received a sedation (1mg (0,2 mL) of acepromazine (Calmivet®, Vetoquinol) intravenously), to avoid any movement and to ensure the good quality of the images. Then, the animal was placed in dorsal recumbency on an artificial insemination table specially designed for use in small ruminants and providing elevation of the hindquarters. Transvaginal ultrasound imaging of the cervix was performed on the animal after an ultrasound coupling gel was applied to the probe to ensure good impedance matching and to facilitate the introduction of the probe through the vagina. For each animal and each measurement, SWE was performed by two expert veterinarians, using a 7-MHz conventional endocavitary ultrasonic probe (SE 12-3, Supersonic Imagine, Aix-en-Provence, France). These data were used to determine the interobserver reproducibility. Both operators were trained to perform the SWE measurement in a standardised pattern and the whole examination did not last more than 5 minutes for each operator.

In the dexamethasone group, 8 ml (2 mg) of dexamethasone (Dexadreson®, Intervet) was administrated intramuscularly after the first SWE image was acquired. Dexamethasone is a reliable and predictable method commonly used for labour induction in sheep. It induces lambing within 30 hours of injection [44–46]. Nothing was administered to induce the labour in the control group.

For all animals, blood was collected from the jugular vein at the beginning of the experiment, just before the first sedation of the first SWE exam (sample T1), and at the end of the experiment, just before euthanasia (sample T2). Blood serum was stored frozen at −20°C until assay.

Following the last examination, the ewes were humanely slaughtered by electrical stunning followed by exsanguination. All the animals were unconscious prior to exsanguination. The uterus was opened, the foetus(es) was (were) immediately euthanised by intra-cardiac injection of a lethal dose of barbiturates (Dolethal®) and a portion of the genital tract including the cervix was collected and fixed in 30% formalin until being processed for histology and optical microscopy analysis.

### Shear waves elastography

Shear Wave Elastography using Supersonic shear imaging (SSI) is an ultrasound-based technique for real-time visualisation of soft tissue viscoelastic properties. This technique has been thoroughly used for the diagnosis of breast cancer [47, 48] or liver fibrosis [49, 50]. SSI utilises acoustic radiation force to generate shear waves remotely, deep in the tissue. Right after the shear waves generation, ultrafast imaging (up to 20000 frames/s) is used to track their velocity [51]. In a homogeneous region of tissue, the Young’s modulus (*E*) or stiffness can be estimated from the SWS (*c*_*s*_) by $E=3\rho c_{s}^{2}$; where *ρ* is the density of the medium through which the waves propagate [52, 53]. This technique is capable of generating plane shear waves of enough amplitude to propagate across the whole image medium, even in highly attenuating media and thus enables the computation of a quantitative elasticity map in strongly viscous media. Many experimental results have shown the stiffness sensitivity of the technique, measuring stiffness values of few Pa [52–54].

The SSI technique enables the mapping of stiffness within the whole cervical area. A great advantage is that it does not require additional set-up and can be performed during routine medical examination. The *Aixplorer* ultrasound system has received *CE* (European Community) clearances for *in vivo* applications to obstetrics. During the whole study, the thermal (TI) and mechanical index (MI) were kept below FDA thresholds. During ultrasound acquisition, so-called “Bmode” imaging, values of TI and MI were 0.2 and 1.4 respectively. In elastography mode, TI and MI where 0.4 and 1.6 respectively.

### Stiffness quantification

**Fig 2:**
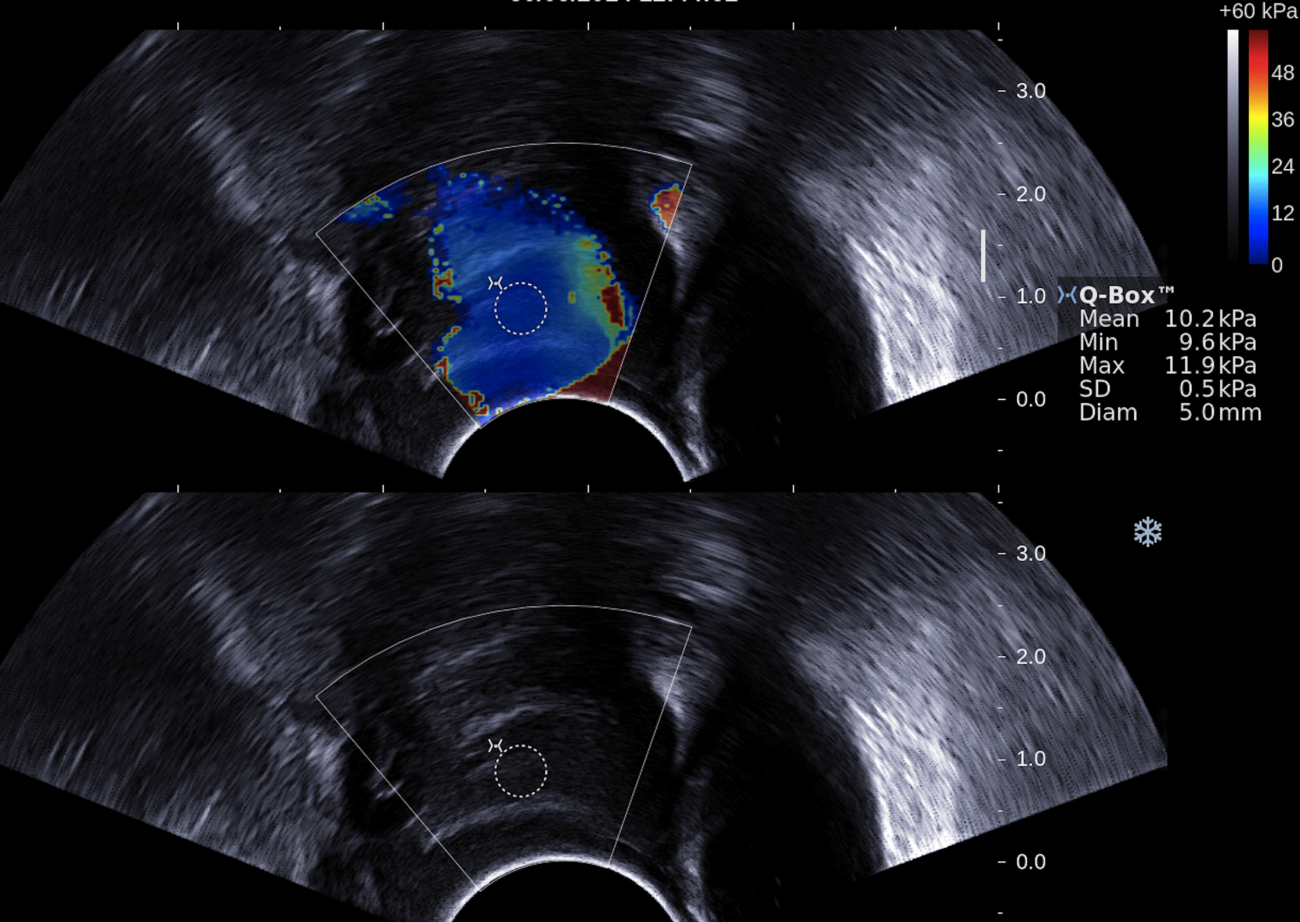
Example of a ROI (white circle) of 5 mm diameter positioned in elastographic image following only anatomical criteria on the conventional B-mode image. Stiffness value estimated inside the ROI: 10.2 kPa ± 0.5 kPa [9.6—11.9] kPa.

The cervical canal of the ewe is characterised by funnel-shaped rings (*N* = 3 − 4), which are not concentrically aligned [55]. The second ring of the cervix was selected to locate the region of interest (ROI) (see Fig 2). Based on the measurements of SWS, the elasticity was quantified in a circular ROI of 5 mm in diameter. The second ring was chosen for several reasons: first because it is a reference point easy to identify in the B-mode image and thus it guarantees quantifying the elasticity always in the same area of the cervix; secondly because it is close enough to the probe to ensure an efficient generation of the shear waves; and finally because it is far enough from both the fetal side and from the vaginal opening to avoid tissue compression from the foetus or from the operator which may generate artificially high stiffness values. The SWE mode of the Aixplorer scanner displays side by side, on the same screen, conventional B-mode and elastographic images. The placement of the ROI within the elastographic image for the SWS and stiffness measurements was determined, using exclusively anatomical criteria, on the conventional B-mode image. This allowed an objective and reproducible positioning of the ROI for velocity and stiffness quantification.

To estimate the intra-observer reproducibility, each practitioner acquired three elastographic images, repositioning the probe every time. The stiffness, calculated in the ROI, and error bars were obtained with the median and standard deviation of these values, respectively.

### Prostaglandin Assay

The normal parturition process is triggered by the rise in fetal cortisol mimicked here by the injection of dexamethasone to the ewe. This rise is associated with the increased placental and endometrial activity of the enzyme prostaglandin H2 synthase (PGHS), which closely correlates with the increase in fetal and maternal plasma prostaglandins [27]. Prostaglandins (PG) are involved in the induction of labour and among the active compounds, Prostaglandin E2 (PGE2) is known to induce cervical relaxation [56].

PGE2 and its metabolites in sheep serum were assayed via the measurement of plasma concentrations of the metabolite Prostaglandin EM (PGEM), using Cayman’s Prostaglandin E Metabolite enzyme-immunoassay (EIA) Kit (Item No. 514531, Cayman chemical). In this test PGE2 and its metabolites are converted to a single and stable derivative (PGEM) that can be easily quantified. PGE2 and PGEM intermediate metabolites from serum (0.5ml) were derived by addition of 0.150 ml of 1 mole/L carbonate buffer and incubated overnight at 37°C. Then 0.200 ml of 1 mole/L phosphate buffer and 0.150 ml of EIA buffer were added to the sample. After acidification to pH 4.00 using 1 mole/L sodium citrate, samples were extracted twice using a mixture of cyclohexane and ethyl acetate (50:50 vol/vol). The organic phase was evaporated under a stream of nitrogen. Samples were then resuspended in the PGEM assay buffer provided in the kit. The PGEM EIA standard was derived following the same procedure. The EIA assay was performed following manufacturer instructions and the EIA plate was read at a wavelength of 410 nm using the EnSpire multimode plate reader (PerkinElmer).

### Histological examination

An axial section was performed by a veterinary pathologist (TL) in the distal part of the cervix in order to locate cervical rings. Once the second ring structure was identified, a 7 mm thick transversal section was performed in this area. Samples were paraffin-embedded and 6 *μm* sections were realised for histological evaluation. Sections were stained using a routine hematoxylin-eosin-saffron (HES). The microscopic evaluation was performed using a light microscope (Eclipse Ni-U, Nikon, Champigny, France) combined with a digital camera (Spot idea camera, Diagnostic Instruments, Sterling Heights, USA). Qualitative analyse of the tissue structures was performed comparing treated and control animals. Using low magnification (X 2), the external perimeter of the cervical mucosa and that of the endocervical lumen were measured on transversal sections. The intra-observer agreement was > 95% as tested by reproducing this measure 5 times on the same sample.

### Second harmonic generation and two-photon fluorescence microscopy

The two-photon microscope used for SHG and 2PF imaging is based on an inverted two-photon excitation fluorescence microscope. A Ti:Sapphire laser beam (wavelength 800 nm, pulse duration 150 fs, repetition rate 80 MHz) is directed to galvanometric scanning mirrors (6215H, Cambridge Technology) and reflected on a dichroic mirror (770DC, Semrock) and then focused onto the sample by an objective lens (Plan Apo Lambda X 20, NA 0.75, Nikon). The obtained lateral optical resolution is about 460 nm. 2PF/SHG images are obtained by scanning the focused beam in the sample plane using galvanometric mirrors, with a typical dwell time of 50 *μ*s per pixel. The fluorescence light is collected in the backward direction by the same objective lens, spectrally separated (488DC Semrock) and further filtered (2PF: HQ540/80M-2P, Chroma, SHG: 400/40 Semrock). The signals are recorded by analog photomultipliers (M9110, Hamamatsu). Typical images are performed in 100 *μ*m x 100 *μ*m regions (300 x 300 pixels), stitched together into a final size of a few millimetres. A Matlab homemade program is used for stitching reconstruction based on an image overlap of 10%. The fixation of the tissue has been found to be unlikely to alter the microstructure addressed by two-photon microscopy images. There is indeed large evidence that fixed and fresh tissues give very similar collagen morphological features in different types of tissues [57–59].

Two-photon microscopy experiments were performed on 6 *μ*m sections realised from the same samples as for histological examination, and thus they approximately correspond to the localisation of the second ring, where the SWE measurements were taken. A 0.17 mm coverslip was placed in order to ensure minimal aberrations in the images. The region of imaging was chosen to cover a size of about 2 mm (two different 2x2 mm^2^ regions per animal were recorded), covering the mucosal chorion, mucous plug and submucosa. Slices obtained for three different nulliparous animals were investigated in the case of control and treatment by dexamethasone, while only one multiparous animal was investigated.

To evaluate the morphological changes observed in the SHG images from different animals and regions, in particular the variations in fibre directionality (waviness) in a SHG image, 2D Fourier transforms (FT) of ROIs of about 60 *μ*m size images were investigated. The shape of the characteristic ellipse represented by the 2D FT of each ROI was evaluated using the Matlab®tool *regionprops*. This tool was implemented on a thresholded 2D FT (using an identical thresholding for all FT images), set at 80% of the maximal (centre) value of the FT image. The ratio R between minor and major axes of the ellipse fitting of the resulting thresholded FT image was calculated based on the measured lengths (in pixels) of the axes of the ellipse that has the same normalised second central moments as the thresholded FT region.

### Statistical analysis

Elastographic measurements were analysed using the F1 LD F1 model of the nparLD function to calculate an ANOVA-type statistic in order to compare the two groups (control and dexamethasone) globally and at each time point using a post-hoc Wilcoxon rank sum test with a Bonferroni-Hochberg correction [60]. Intra and interobserver repeatability of measurements were assessed by Bland-Altman analysis [61]. The Bland-Altman plot of the average against the differences of the two measurements was performed and the 95% limits of agreement were calculated for each measurement to examine the agreement and bias between the same and the two different examiners for each measurement.

The results of the PGE2 assay were analysed with a Kruskal-Wallis non-parametric test. For the 2D FT results of the SHG images, a statistical analysis was performed on 5 to 10 ROIs measured in two different animals per case (control and treated), for two different regions (mucosal chorion and submucosa). One-way ANOVA tests (Levene?s Test of Homogeneity of Variance, Tukey HSD paired comparison) were performed between the control/treated categories in both regions. A similar statistical analysis was performed on the maximum intensity measured in each ROI image, taken as the average obtained between the 10 pixels of highest intensity in an image.

Statistical analyses were carried out using *R* software (www.r-project.org/, version i386 2.15.2). All results are expressed as median ± standard deviation. Data were considered statistically significant for *p* < 0.05.

## Results

The characteristics of the animals are presented in Table 1. Cervical elastography could be performed in all cases (n = 9). A total of 378 ROIs were analysed for stiffness evaluation (9 animals x 7 measurements x 2 operators x 3 images/operator). During the last examination (24 hours after first examination), one animal had to be removed from the study because no reliable measurement of cervical stiffness was obtained due to movements of the animal (*n* = 6 images, i.e., 1.6% of the total number). Images with strong artefacts, for which standard deviation within the ROI were higher than 30% of the nominal value, leading to apparent strong heterogeneities in the stiffness were rejected too (*n* = 2 images, i.e., 0.53% of the total number). No ewes showed any obvious sign of contraction or behavioural preparation for parturition (nesting behaviour, restlessness).

**Table 1:** Characteristics of the study population.

Characteristic	Control group	Dexamethasone group
Total population (number)	4	5
Gestational age (days)	127	127
Nulliparous (number)	3	4

**Fig 3:**
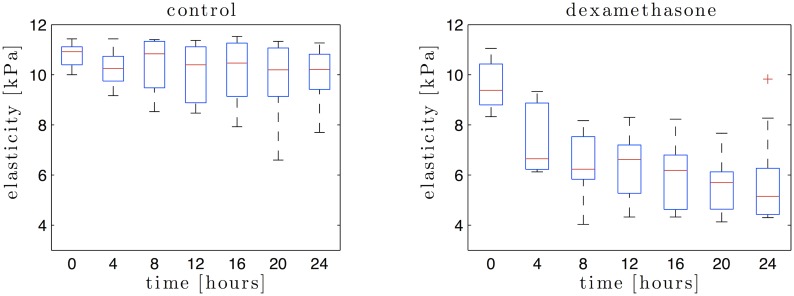
Cervical stiffness measured for the control (left) and dexamethasone group (right) in the 7 examination performed over 24 hours.

Fig 3 shows the distribution in form of box plots for each measurement time, and for both control and dexamethasone group. In the dexamethasone group, stiffness values are significantly lower than the values measured in the control group. The minimum stiffness values correspond to the two last examinations (measurements taken after 20 and 24 hours). Since elasticity, *E*, is estimated from SWS, the same trends are found for both. Here, only stiffness values are shown. The mean initial stiffness values were not significantly different between groups (10.79 kPa ± 0.48 kPa and 9.54 kPa ± 0.92 kPa, *p* = 0.176, for the control and dexamethasone group, respectively). In the dexamethasone group, stiffness decreased significantly with cervical maturation time, with a steep decrease within the first 4 hours (7.43 kPa ± 1.39 kPa at 4 hours, *p* < 0.05) followed by a plateau. On the contrary, non significant trend was observed in the control group (average last stiffness value is 10.00 kPa ± 1.18 kPa).

At 24 hours, the last SWS and consequently the average last stiffness value were significantly lower in treated versus non-treated animals (1.826 m/s ± 0.627 m/s and 10.00 kPa ± 1.18 kPa in control ewes versus 1.291 m/s ± 0.523 m/s and 5.00 kPa ± 0.82 kPa treated ewes, *p* < 0.05). A second order fit could be obtained for the stiffness versus cervical maturation time in the control (*R* ^2^ = 95%) and dexamethasone group (*R* ^2^ = 83%). The dexamethasone group shows a significant higher negative slope (0.0103*t* ^2^ − 0.3844*t* + 9.2147) than the control group (0.0014*t* ^2^ − 0.0648*t* + 10.7016).

When data were analysed using only the nulliparous animals, results were the same, and the elastographic profiles of the multiparous ewes closely matched that of the nulliparous ones.

**Fig 4:**
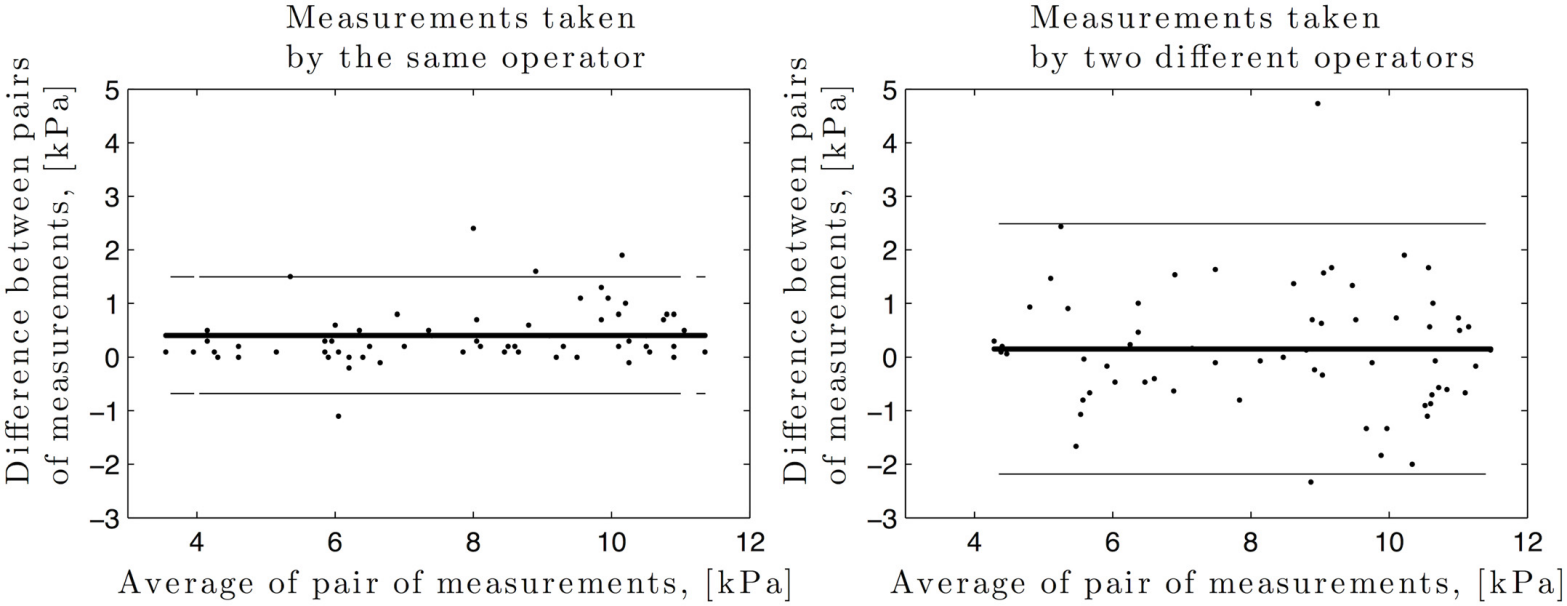
Bland-Altman plots demonstrating degree of concordance between pairs of cervical elastography measurements obtained by the same (left) and by two different operators (right). Mean difference (solid line) and 95% limits of agreement (dashed lines) are shown.

Bland-Altman plots, demonstrating the degree of concordance between pairs of measurements made by the same and by two different observers, are given in Fig 4. There was no significant bias in any case because the difference between measurements remained stable as the average increased. The mean and standard deviation of differences appeared to be constant throughout the range of measurements for all comparisons. Note that the only few points where both operators do not agree are scattered between times and animals.

**Fig 5:**
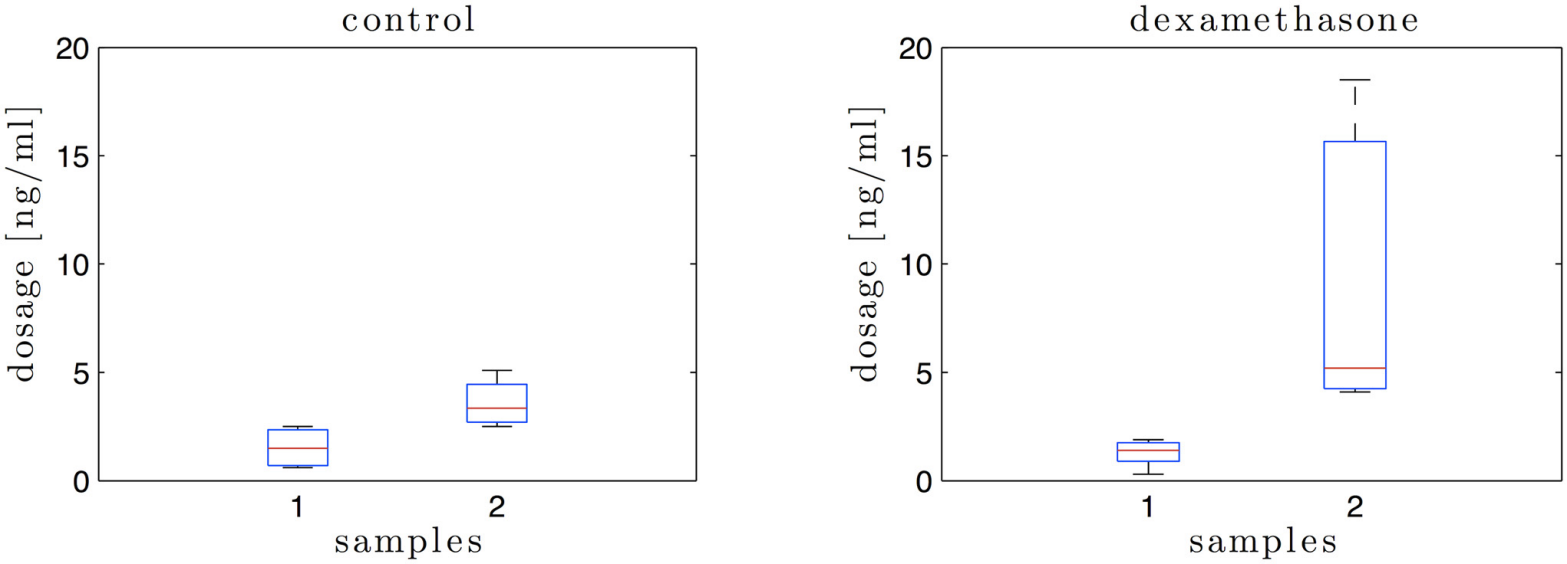
PGE2 and its metabolites in sheep serum just before the first elastographic exam (sample 1) and at the end of the experiment just before euthanasia (sample 2), assayed via its metabolite PGEM with Cayman’s Prostaglandin E Metabolite EIA Kit (Item No. 514531, Cayman chemical).

Plasma PGE2 concentrations were not significantly different between groups at the beginning of the experiment. In contrast, as shown in Fig 5, there was a sharp and significant increase in PGE2 24 hours following dexamethasone treatment (*p* < 0.01), whereas values were not significantly different in the control group.

**Fig 6:**
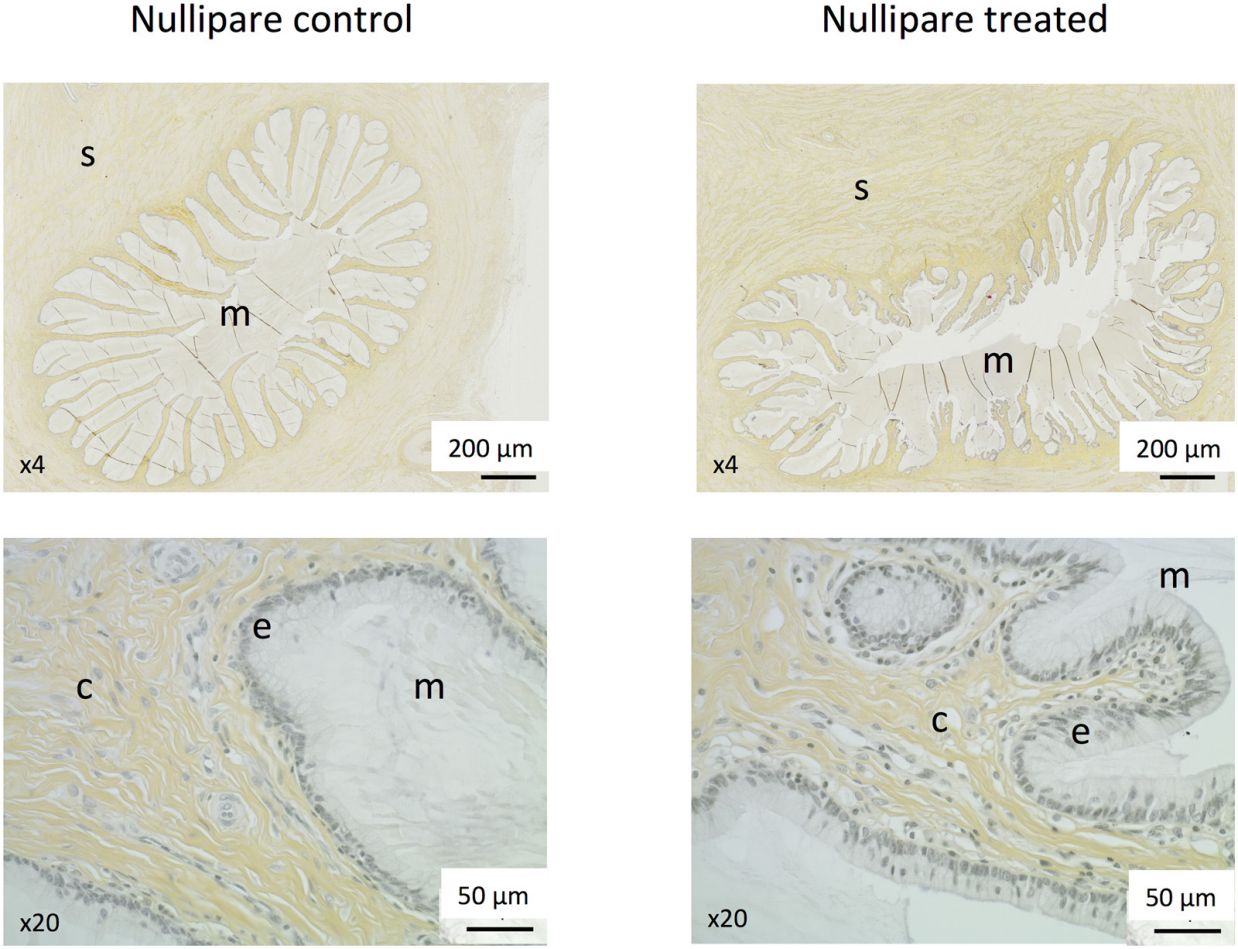
Complete histological evaluation of the cervix. Mucous plug (m), epithelial cells (e), mucosal chorion (c), submucosa (s).

**Fig 7:**
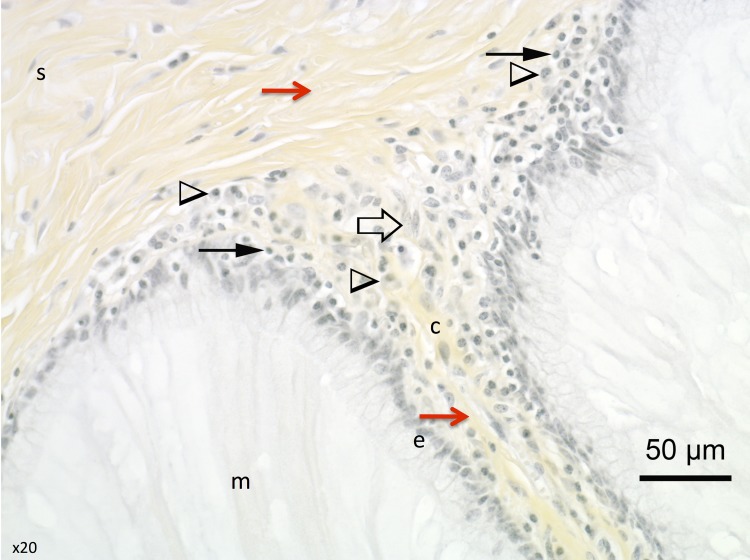
Structures identified by histological evaluation in the cervical tissue. Mucous plug (m), epithelial cells (e), mucosal chorion (c), submucosa (s). Collagen fibres: red arrow; Macrophages: arrowhead; lymphocytes: solid arrow; fibroblasts: empty arrow.

**Fig 8:**
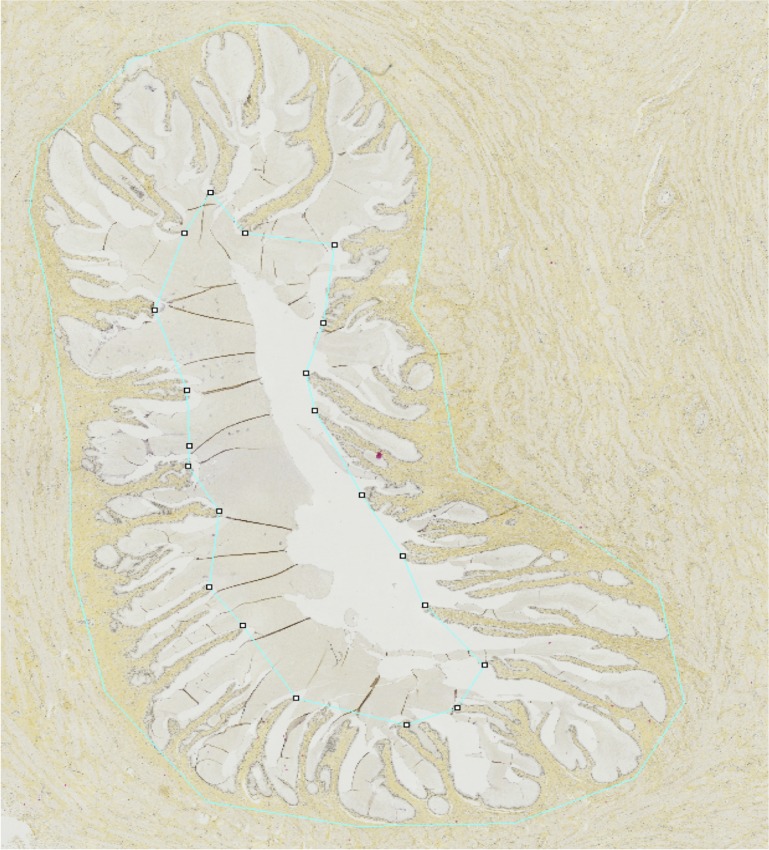
Measurement of the external diameter of the cervical mucosa (green line).

During the complete histological evaluation of the cervix, no systematic difference was observed between untreated and treated animals (see Fig 6). After hematoxylin-eosin-saffron (HES) staining, collagen fibres of the mucosal chorion and the submucosa appeared very similar between animals. Some inflammatory cells were present just beneath the mucosa and were consistently more numerous in treated animals compared to untreated ones although some high inter-individual variability precluded any definitive conclusion. The different structures identified by histological evaluation are shown in Fig 7. External diameter of the cervical mucosa tended to be higher in treated animals compared to untreated ones, respectively 7544 ± 337 and 6805 ± 327 *μ*m, indicative of the relaxed gross appearance of the cervix in these animals (Fig 8). No difference in the mucosal fold height was depicted between animal groups.

**Fig 9:**
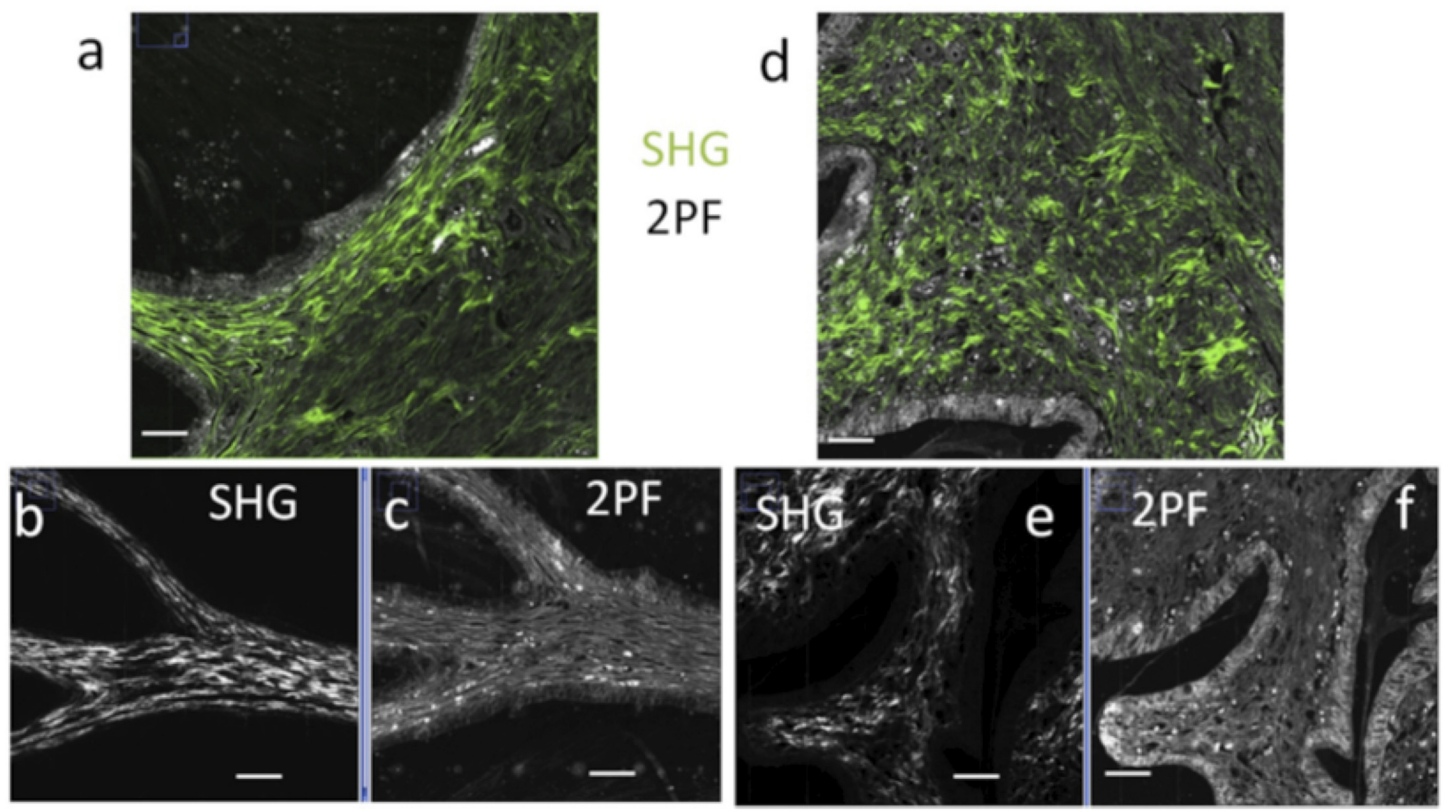
SHG/2PF images. (a-c) control and (d-f) treated samples imaged in two photon microscopy, in the mucosal chorion/submucosa region of fixed slices. (a,d) Composite 2PF(grey)/SHG(green) image (scale bar: 100 *μ*m). Separated SHG/2PF are shown in (b,c) and (e,f) inside the mucosal chorion (scale bar: 100 *μ*m).

**Fig 10:**
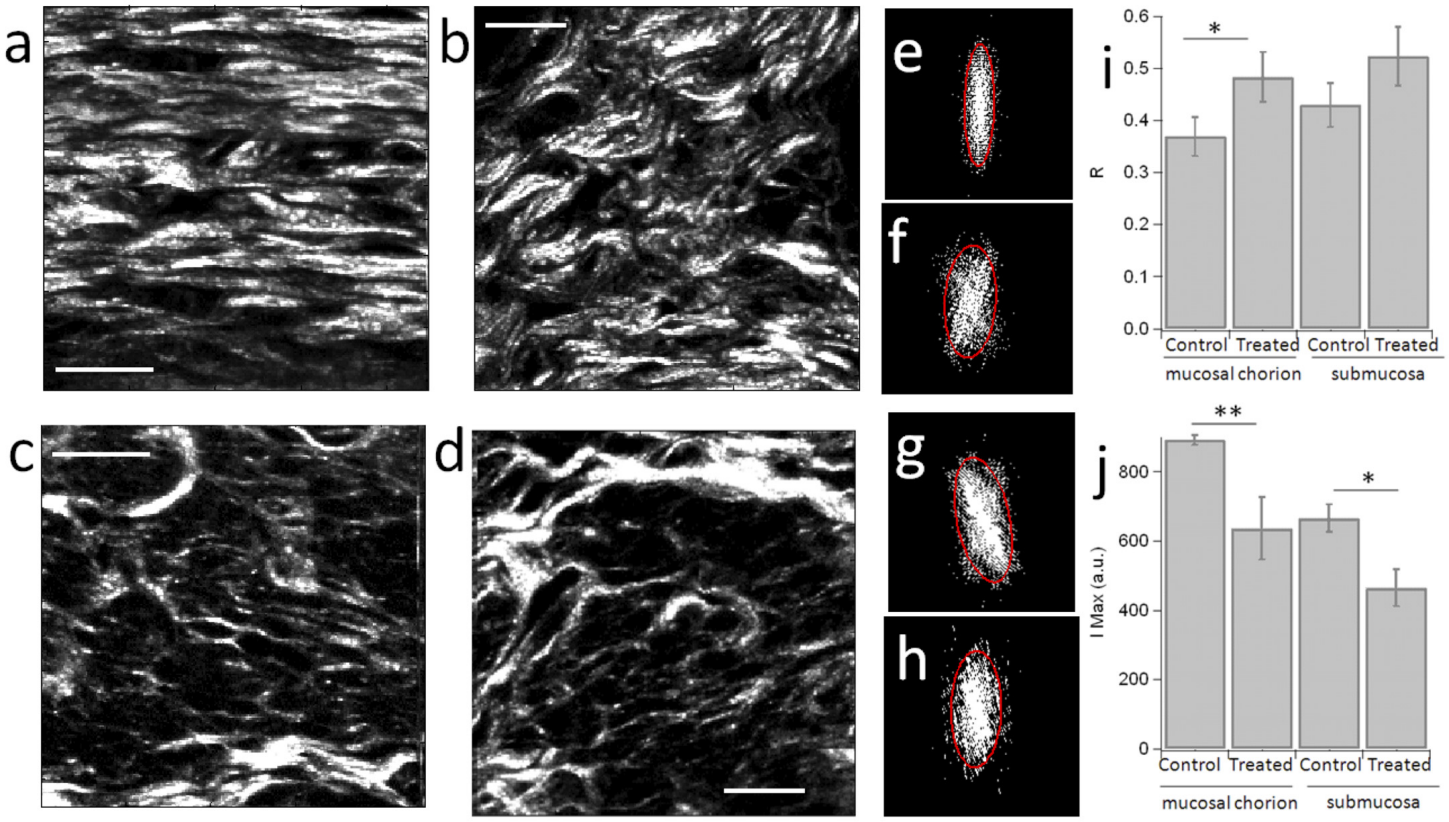
SHG zoomed images. Typical SHG zoomed images in the mucosal chorion region of (a) control and (b) treated samples, and in the submucosa region of (c) control and (d) treated sample. Scale bars: 20 *μ*m. SHG ROI images are intentionally contrasted in order to enhance the visibility of the morphological differences between the control and treated samples. Corresponding 2D FT images, thresholded at 80% of their maximal intensity, are shown in (e-h) for (e) control/mucosal chorion (maximum intensity = 971 counts, R = 0.28), (f) treated/mucosal chorion (maximum intensity = 737 counts, R = 0.39), (g) control/submucosa (maximum intensity = 581 counts, R = 0.42), (h) treated/ submucosa (maximum intensity = 412 counts, R = 0.43). Average values and standard deviations (plotted error bars) are given for 5-10 regions, measured from two different animals (* = significant at 5% level; ** = significant at 1% level).

2PF mucosal chorion and SHG imaging were performed simultaneously in 2 mm size regions covering the chorion, mucous plug and submucosa, with a spatial resolution of about 460nm (Fig 9). Control and treated samples showed several differences, especially in the mucosal chorion, with consistency between different animals. Fig 10 shows zoomed images in control and treated samples. SHG images in the control cases show well defined directional structures that are very reproducible from one region to another (Fig 10a and 10b). Whereas, the treated case shows more disorganised structures, at the scale of a few tens of *μ*m up to 100 *μ*m (Fig 10c and 10d).

## Discussion

Cervical stiffness is continuously decreasing during pregnancy until the onset of labour, when dramatic changes in consistency lead to opening the cervix and allowing the foetus to pass. In the control animals, no statistical difference in cervical stiffness was observed at any time in this study. Stiffness values matched those measured before the dexamethasone injection in the treated group. In contrast, cervical stiffness decreased significantly within 4 hours after induced cervical maturation by dexamethasone injection. Previous studies have reported that cervical tissue is significantly softer at the end of the third trimester than in early pregnancy [18, 22, 23].

The cervix undergoes important changes during cervical ripening. This process involves catabolic processes leading to collagen degradation, inducing modifications of the collagen network, its geometrical configuration and mechanical properties within the cervical tissue. Morphological features of the cervical collagen matrix changes during pregnancy encompass its transformation from an alienable fibrous matrix to an amorphous hydrated matrix capable of undergoing large distention [29]. These changes at the microstructure have been reported previously using nonlinear images, however its relation with the tissue mechanics at the macroscale is still not fully understood. In this study, combining SWE and two-photon excitation microscopy, we provided a preliminary insight about this connection. Therefore, this study did not aim to describe the cervical remodelling, but set a relation between cervical mechanics and collagen network. SHG images exhibit collagen fibres assemblies with characteristic shapes similar to previous results on mice and humans [28, 62, 63]. From a qualitative observation, while the submucosa submucosa region does not show significant morphological differences in the collagen fibres between the control and treated cases, the mucosal chorion is seen to undergo the strongest modifications. Fibres size and waviness as well as pore sizes are conserved throughout the mucosal chorion, with a more wavy and disorganised structure in treated samples (Fig 9d and 9e). Zoomed images in the control samples shows well defined directional structures that are very similar from one region to another and from one animal to another. In the mucosal chorion, while in the control sample, the fibres are seen to be well aligned and parallel (Fig 10a), the treated case shows more disorganised structures, at the scale of a few tens of *μ*m up to 100 *μ*m (Fig 10b). Note that there is no significant change in the fibre diameter, however fibres density is seen to slightly decrease in treated samples, with a visible change in waviness. In the submucosa region, for both control and treated samples, fibres are much more disorganised and heterogeneous in diameter, waviness and length (Fig 10c and 10d). In order to quantify the shape changes observed in the SHG images, 2D Fourier transform (FT) analysis of 5 to 10 ROI images size per region (chorion mucosa, submucosa) was performed in two different slices (e.g. two different animals). The size of these regions (typically 60 *μ*m) was intentionally chosen such as to exhibit representative homogeneous structures, which were persistent in shapes over about a mm scale in the tissue SHG image. A 2D FT from aligned, parallel fibres results in an elliptical shape which orientation reflects the average direction of the fibres (given by its perpendicular direction), with a width that reflects the extent of variations of the fibres directions along this average direction. This width is quantified by the ratio R between the ellipse short axis length and its long axis length. Fig 10e–10h depicts typical ellipses obtained from thresholded 2D FT analysis (see Methods section) for the SHG images shown in Fig 10a–10d. A small R value means that fibres are very parallel to each other, while a high R value reflects more random orientations of fibres present in the image, including enhancements of waviness in the fibres shapes. The results (Fig 10i) show that R decreases from the control to the treated cases, both in the chorion mucosa and submucosa regions, with a more pronounced effect in the chorion mucosa. This reflects the loss of fibre directionality in the submucosa, as visible by the apparition of waviness. The treatment thus induces a clear disorganisation of collagen fibres at a scale of tens of *μ*m (scale of the observed waviness of fibres in Fig 10b). In addition the SHG efficiency of collagen fibres is also seen to depend on the region and case. Maximum intensities (averaged over the 10 brightest pixels) were compared in the same ROI regions are previously analysed by 2D FT. The result, depicted in Fig 10j, shows that the collagen SHG intensity significantly decreases from the control to treated region in both chorion mucosa and submucosa regions. These results are consistent with a more organised structure in the control chorion mucosa regions at the microscopic scale, where more parallel fibres lead to a more efficient build up of the coherent SHG signal and result in a higher intensity. This intensity effect also most probably reflects the loss of collagen density at a micrometric scale. This microscopic scale observation is consistent with the degradation of the circumferential ring of collagen fibres observed in the cervix at the late stages of pregnancy in human [28]. Overall, the quantitative changes in the mechanical properties probed by elastography seem to correspond to qualitative structural changes observed at the microscopic scale. This is in the agreement with the study [64], and seems to enforce the idea that straight and organised structures would favour more stiff mechanical properties. The relation between sub-micrometric resolution scale morphology and stiffness has been already evoked in previous work in other tissues such as collagen-rich tendons [65]. Similar results of cervix SHG imaging have been more recently obtained using endomicroscopy [62] and combined with B-mode ultrasound imaging and quantitative ultrasound (QUS) measurements, which corroborate it [63]. This supports the strong relation between collagen organisation and tissue mechanical properties, as also recently shown in various studies [65–68].

2PF images show complementary information (Fig 9c and 9f). First, they show in clear contrast the contour of epithelial cells. They also exhibit fibrillar structures that are in close connection with collagen fibres. These fibres do not necessarily spatially overlap with collagen fibres, and could be assigned partially to elastin, with a structure visibly affected by the treatment since destructuration appears. Even though the precise chemical identification of these structures is not known, their integrity is clearly seen to be affected in the case of treated animals. Both collagen and 2PF-active fibrillar structures would then be closely related to the mechanical properties of cervical tissues that are perceptible in elastography.

The morphology of collagen does not show any strong differences between control and treated animals in histology results, in contrast to optical microscopy images, probably due to the different origin of the contrast and to the scale of observation which is larger in histology observations. Histology results show that the external diameter of the cervical mucosa increases in treated animals compared to control ones (Fig 8). This is in agreement with previous observations. It has been reported that the circumferential ring of collagen fibres presents in the cervix at early term disappear at later stages of pregnancy, close to delivery [28]. This phase of cervical remodelling gives the cervix the ability to dilate in response to uterine contractions of labour. The B-mode images presented in this study are consistent with this occurrence of remodelling. In the control group, as well as for the first measurement of the dexamethasone group, it is possible to identify some well defined structures in the rings that characterise the cervix of sheep. As soon as the dexamethasone starts to operate, these structure become diffused, until they practically disappear in the last measurements.

It is known that dexamethasone reaches its maximum effect between 2 and 3 hours following injection [69], and this reflected by the fact that the highest drop in elasticity is observed in the first measurement after injection. The second order fit performed in the treated group confirms that dexamethasone effects are higher during the first hours.

Previous studies performed in humans suggest that ripened cervices are more like each other than are the unripened ones [25]. This can be consistent with the fact that in the animals treated by dexamethasone, elasticity measurements were not significantly different between nulliparous and multiparous animals, whereas the control group presented significant differences. However, in this study, no trend was observed in the dynamic range of stiffness. In sheep, fetal endogenous cortisol secretion rise only in the last two weeks of gestation whereas in humans, the rise starts from mid-pregnancy [70] and this may be the reason for this difference between observations in the present study and that in women.

The objective of this study was to explore the SWE method’s feasibility and to describe the elastographic patterns of the cervical maturation. A comprehensive quantitative assessment of cervical ripening requires the calculation of the elastic moduli. The main limitation of this study is related to the assumptions made for the assessment of stiffness from the shear wave speed values, namely a locally homogeneous, isotropic and semi-infinite medium. To estimate the elasticity, the SSI technique relies on the assumption that the medium is locally homogeneous, isotropic and semi-infinite. However, at the beginning of this experiment, before cervical maturation was induced by dexamethasone, collagen fibres may present a high orientation and thus these conditions might not have been satisfied. The cervix is heterogeneous, anisotropic and only a few times larger than shear wavelengths that are characteristic in SSI. However, all of the statistical tests have been done very carefully, and apply to the shear wave speed values as well as to the stiffness values. The ROI’s assume small homogeneous areas and they allow the assessment of the ability of SSI to distinguish between ripened and unripened cervix by quantifying the elasticity. Further studies are required to judge the effect of the above parameters and to compare the findings in women. The changes in the mechanical properties of the tissue (measured with SWE) induced by the dexamethasone were associated with changes in the micro-structural organisation of the collagen matrix (measured with SHG and 2PF). This is consistent with previous findings in the liver [71] and in the prostate [72]. Future works should focus on a longitudinal study to deeply explain the link between stiffness and the cervical remodelling.

Shear waves elastography measurements using SSI technique were highly reproducible in all the cases. In order to obtain reproducible measurements, the pressure applied on the cervix by the sonographer with the probe had to be minimal, and the ROI have to be placed away from the surface of the probe. The probe was inserted and once the rings could be visualized, the probe was gently pulled back towards the operator in order to make sure that no compression was applied, using B-mode image as an indicator of tissue deformation. Moreover, the placement of the ROI at the second ring, i.e., away from the surface of the probe, aimed at avoiding any bias due to compression.

## Conclusions

The principal findings of the study are that, for first time, according to our knowledge, a quantitative image of the cervix was assessed by using SWE technique. The reproducibility and sensitivity of the technique has been demonstrated. We have shown that the stiffness measurements obtained by the same and by two different observers are reliable and reproducible. Stiffness of the uterine cervix changes throughout the maturation process induced by the dexamethasone injection. In general, the control group (unripened cervix) presents the highest stiffness, and the lowest stiffness values correspond to a measurement closer to delivery in the labour induction group. In addition, the maturation process was validated by a significant increase in PGE2 24 hours following dexamethasone treatment and by changes in the micro-structure of the tissue as observed by histological analysis and nonlinear imaging. Finally, relevant insight in the cervical microstructure was provided by the combination of SHG and 2PF, which allows different scales of investigation. These addressed changes at the microscopic scale during cervical remodelling may be partly responsible for the mechanical properties measured by elastography.

Objective measurements of stiffness reflect histological changes that could provide a measure of cervical ripening, and shear waves elastography may be a valuable method to objectively quantify the cervical stiffness. Standardisation of the cervical properties observed on elastography during pregnancy may help to guide the artificial labour induction and to diagnose preterm birth. Further studies are needed to relate shear wave propagation with histological and endocrine modifications in the cervix. For this purpose, animal models are necessary as they provide a means to generate physiologically relevant data that can then be evaluated in humans.

## Supporting Information

S1 Fig Examples of elastographic images. Example of a ROI (white circle) of 5 mm diameter positioned in elastographic image following only anatomical criteria on the conventional B-mode image.
(ZIP)
