# Supplementary figures and images for: In Vivo Evaluation of Cervical Stiffness Evolution during Induced Ripening Using Shear Wave Elastography, Histology and 2 Photon Excitation Microscopy: Insight from an Animal Model
Source: PLoS One. 2015 Aug 28;10(8):e0133377. doi: 10.1371/journal.pone.0133377 (PMC4552804; doi:10.1371/journal.pone.0133377)

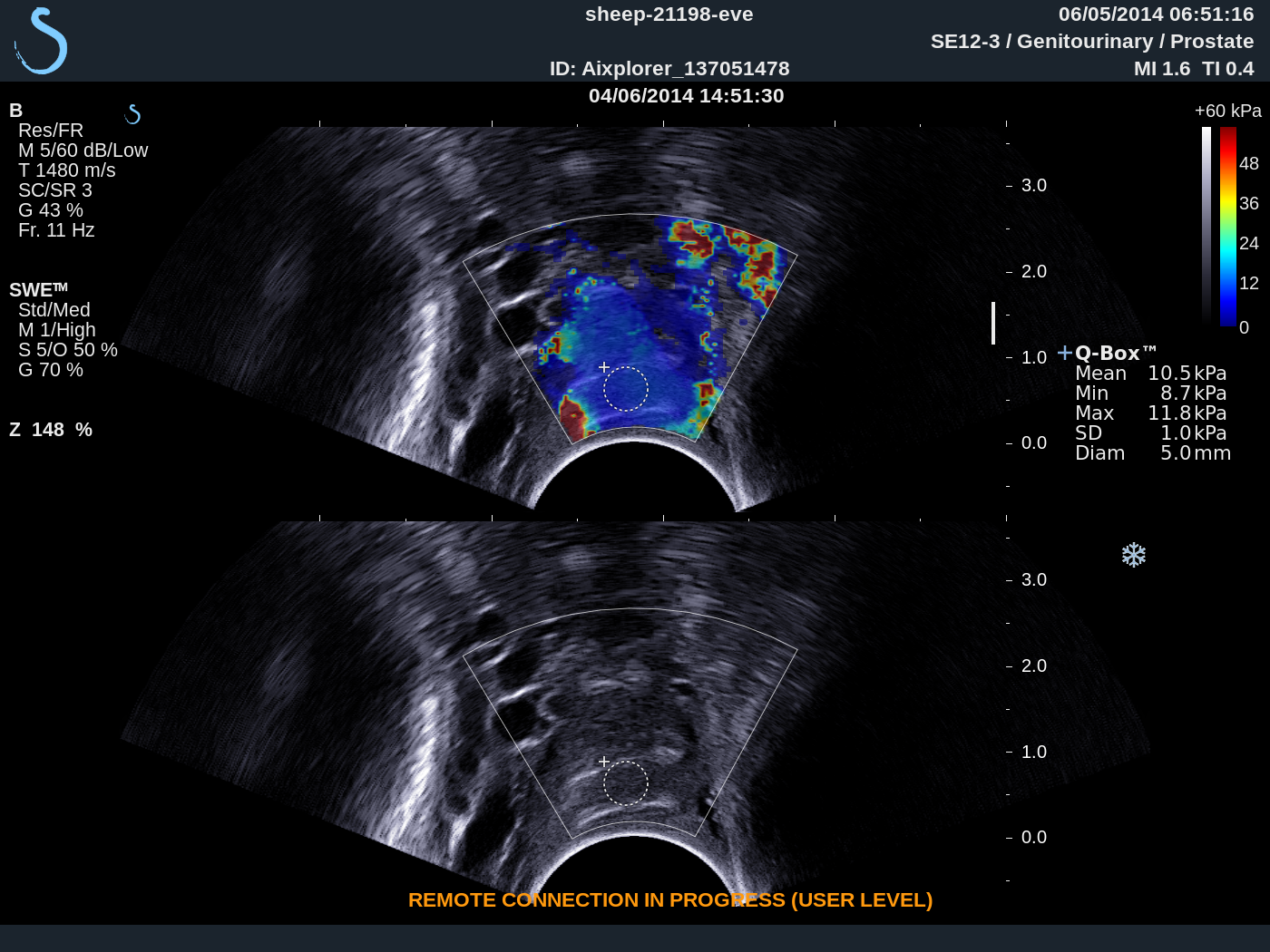

Supplement: S1 Fig — Example of a ROI (white circle) of 5 mm diameter positioned in elastographic image following only anatomical criteria on the conventional B-mode image. (ZIP) [file pone.0133377.s001.zip › S1 Fig/2014_06_04_145126_571.png]

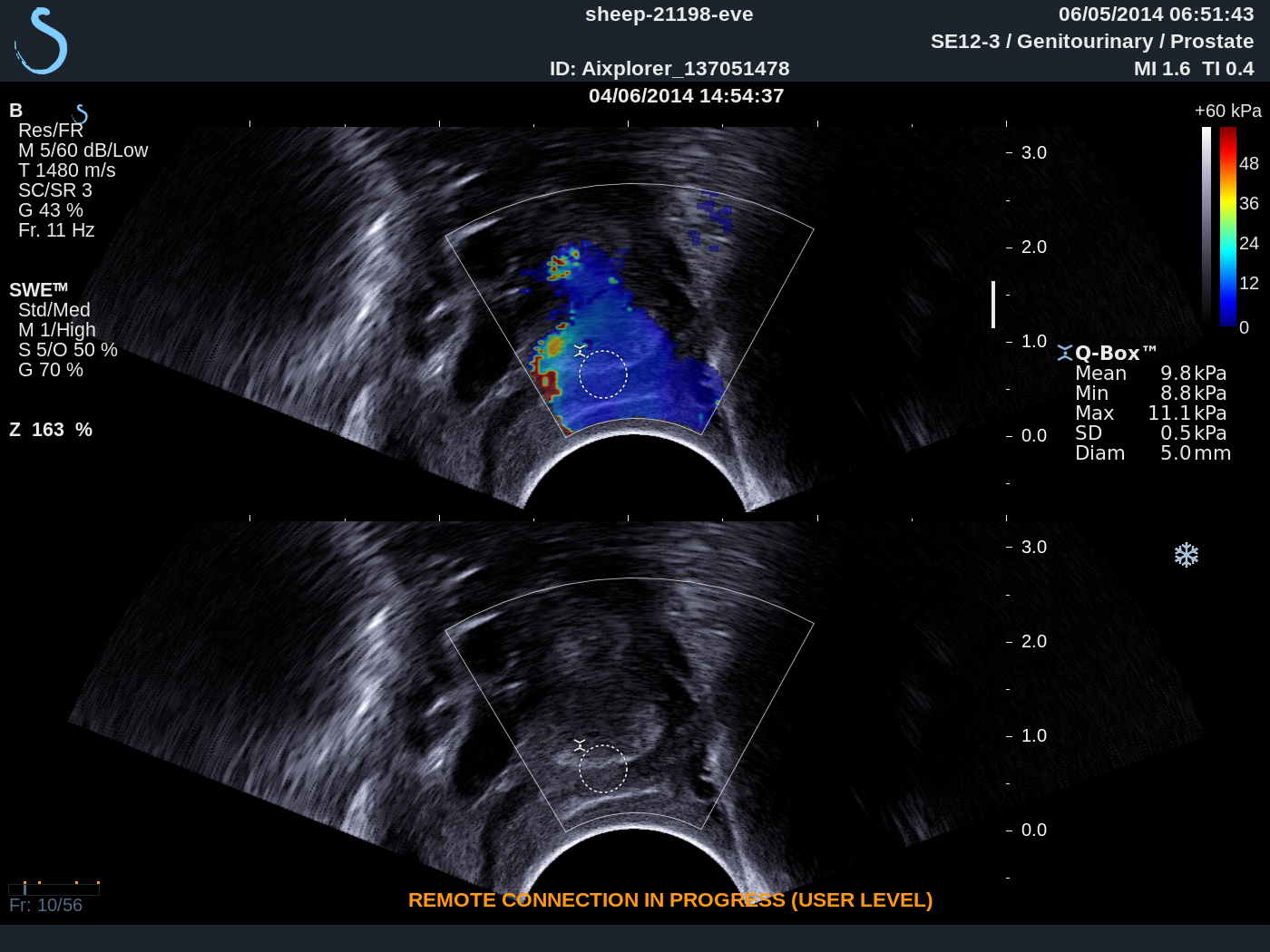

Supplement: S1 Fig — Example of a ROI (white circle) of 5 mm diameter positioned in elastographic image following only anatomical criteria on the conventional B-mode image. (ZIP) [file pone.0133377.s001.zip › S1 Fig/2014_06_04_145437_420.png]

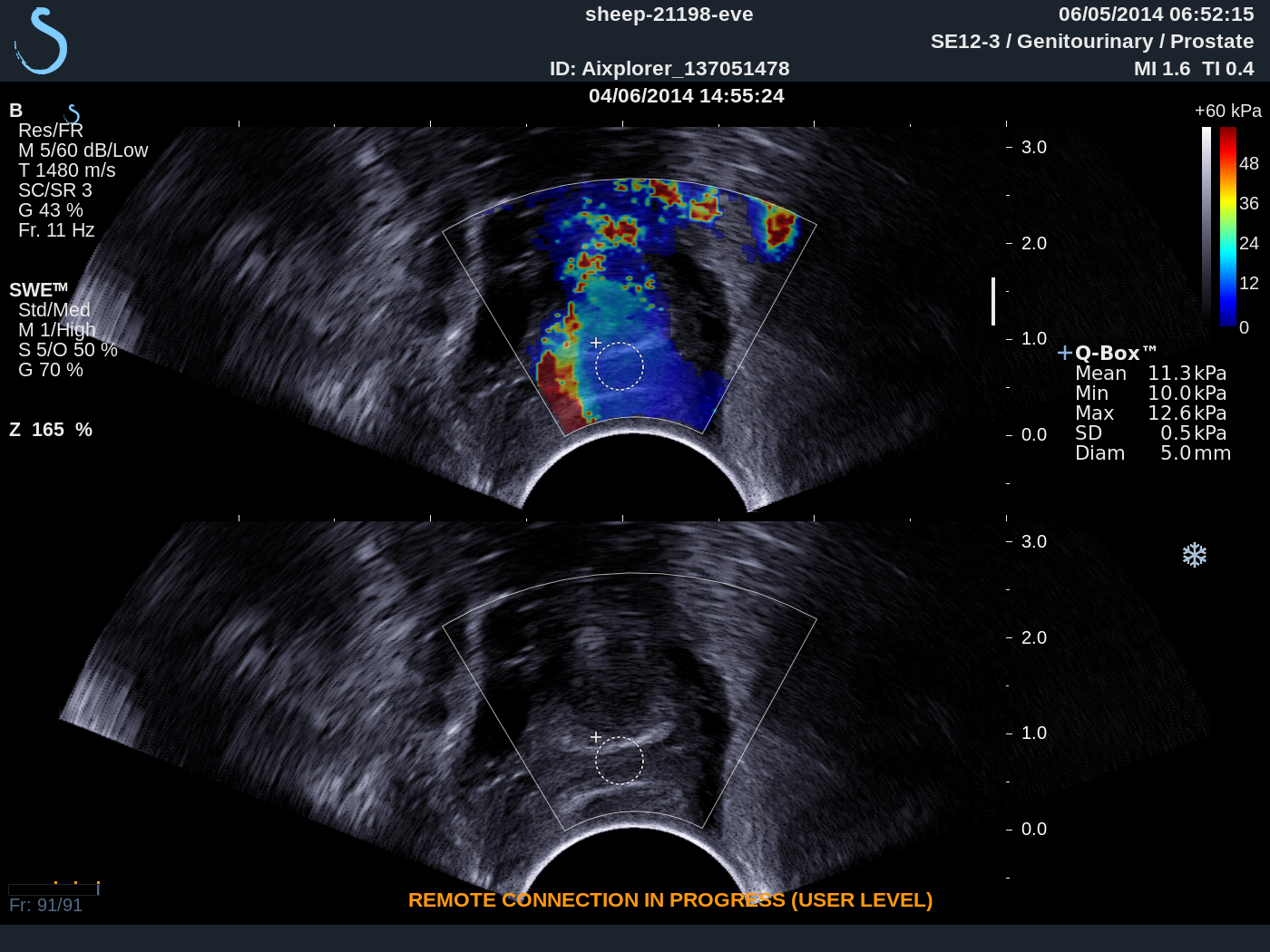

Supplement: S1 Fig — Example of a ROI (white circle) of 5 mm diameter positioned in elastographic image following only anatomical criteria on the conventional B-mode image. (ZIP) [file pone.0133377.s001.zip › S1 Fig/2014_06_04_145523_696.png]

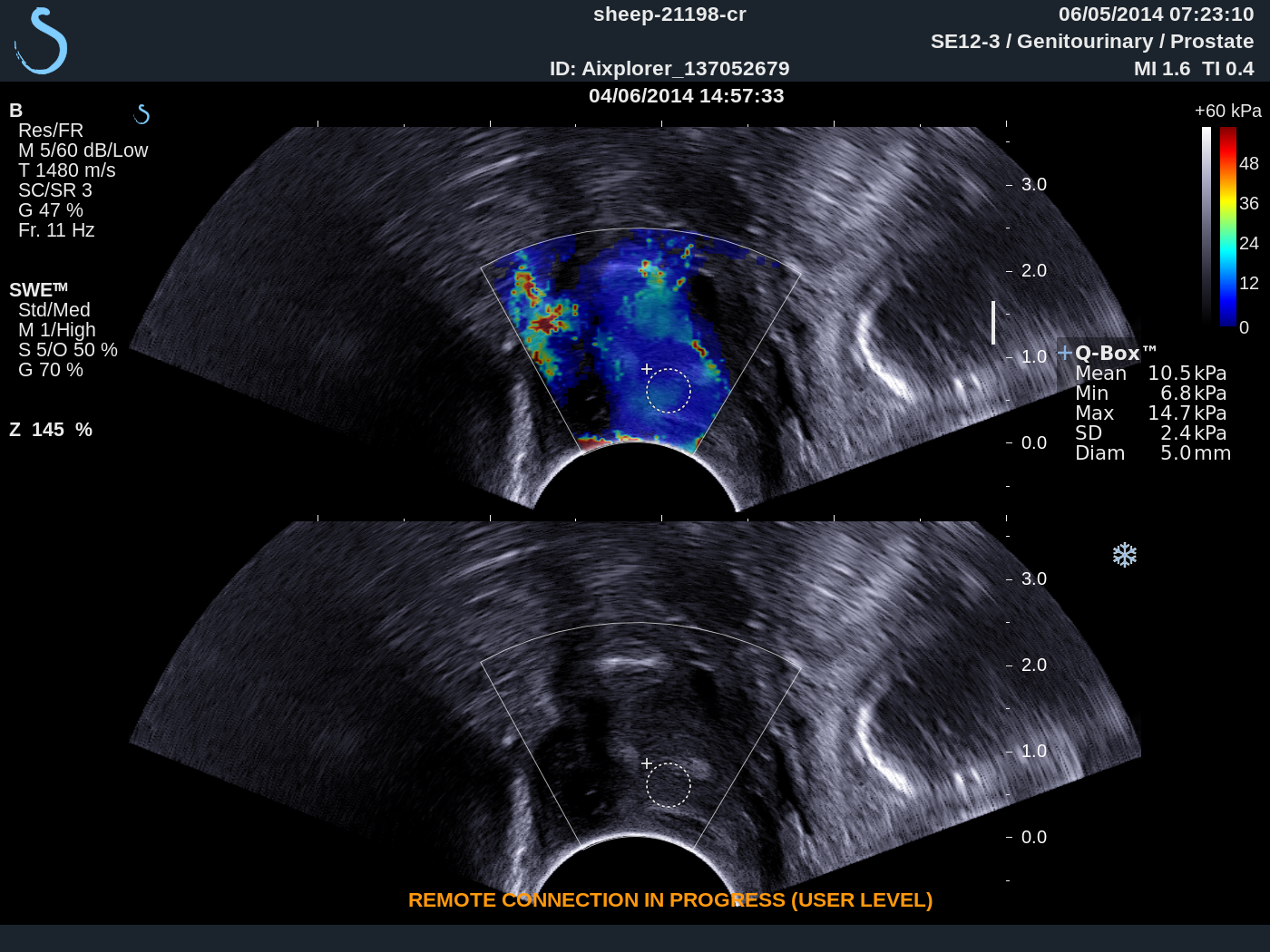

Supplement: S1 Fig — Example of a ROI (white circle) of 5 mm diameter positioned in elastographic image following only anatomical criteria on the conventional B-mode image. (ZIP) [file pone.0133377.s001.zip › S1 Fig/2014_06_04_145733_614.png]

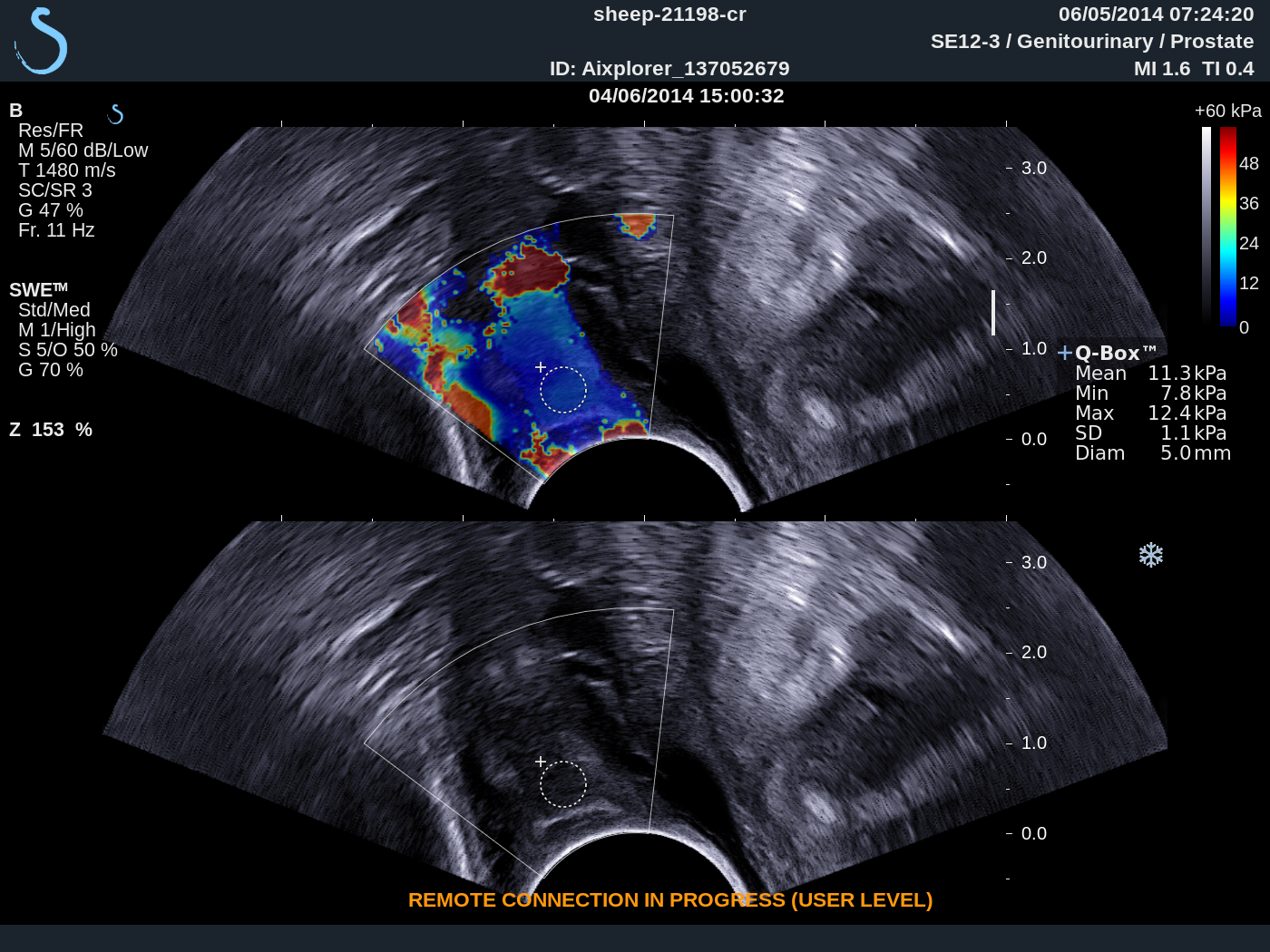

Supplement: S1 Fig — Example of a ROI (white circle) of 5 mm diameter positioned in elastographic image following only anatomical criteria on the conventional B-mode image. (ZIP) [file pone.0133377.s001.zip › S1 Fig/2014_06_04_150032_376.png]

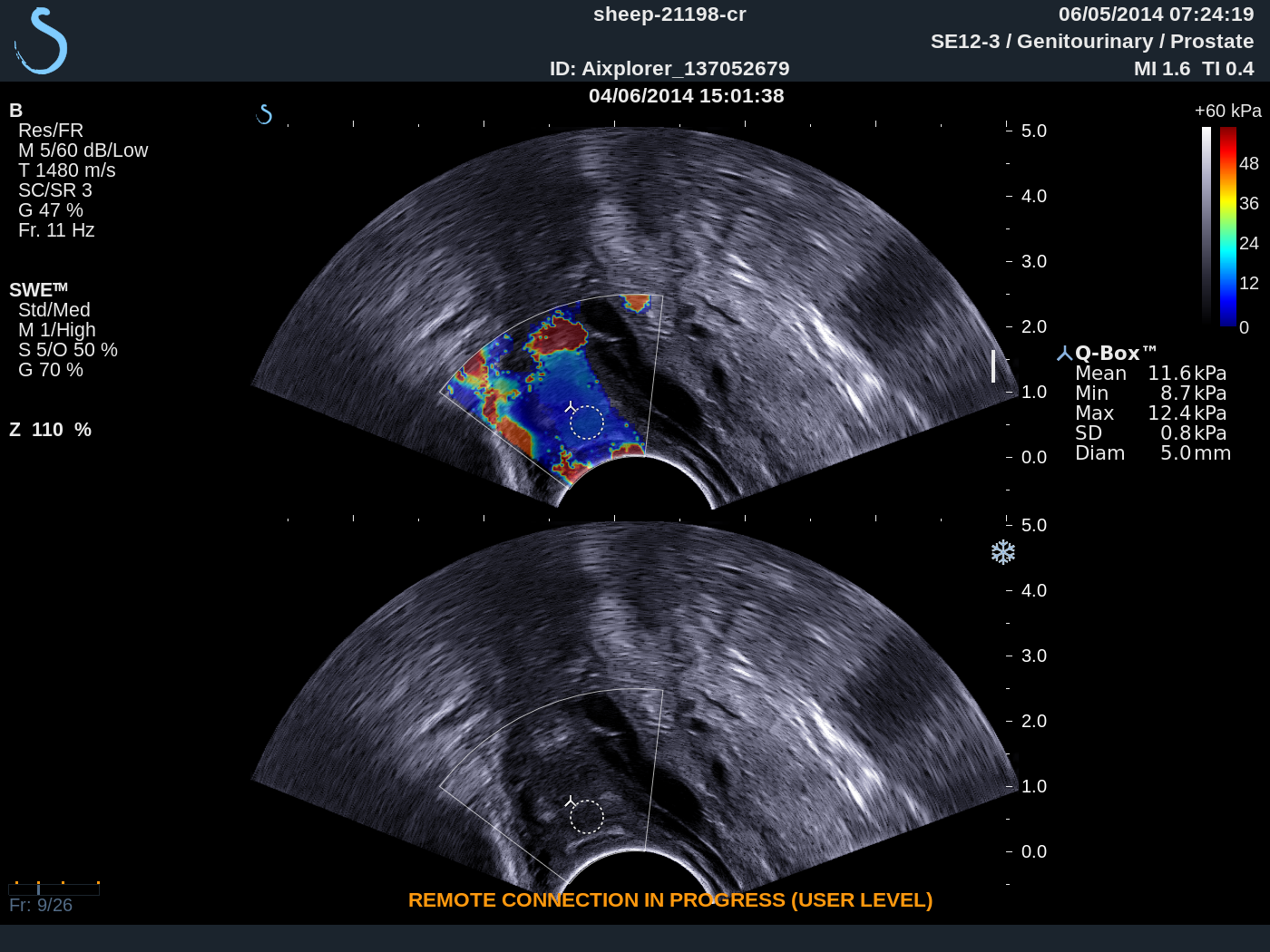

Supplement: S1 Fig — Example of a ROI (white circle) of 5 mm diameter positioned in elastographic image following only anatomical criteria on the conventional B-mode image. (ZIP) [file pone.0133377.s001.zip › S1 Fig/2014_06_04_150138_115.png]

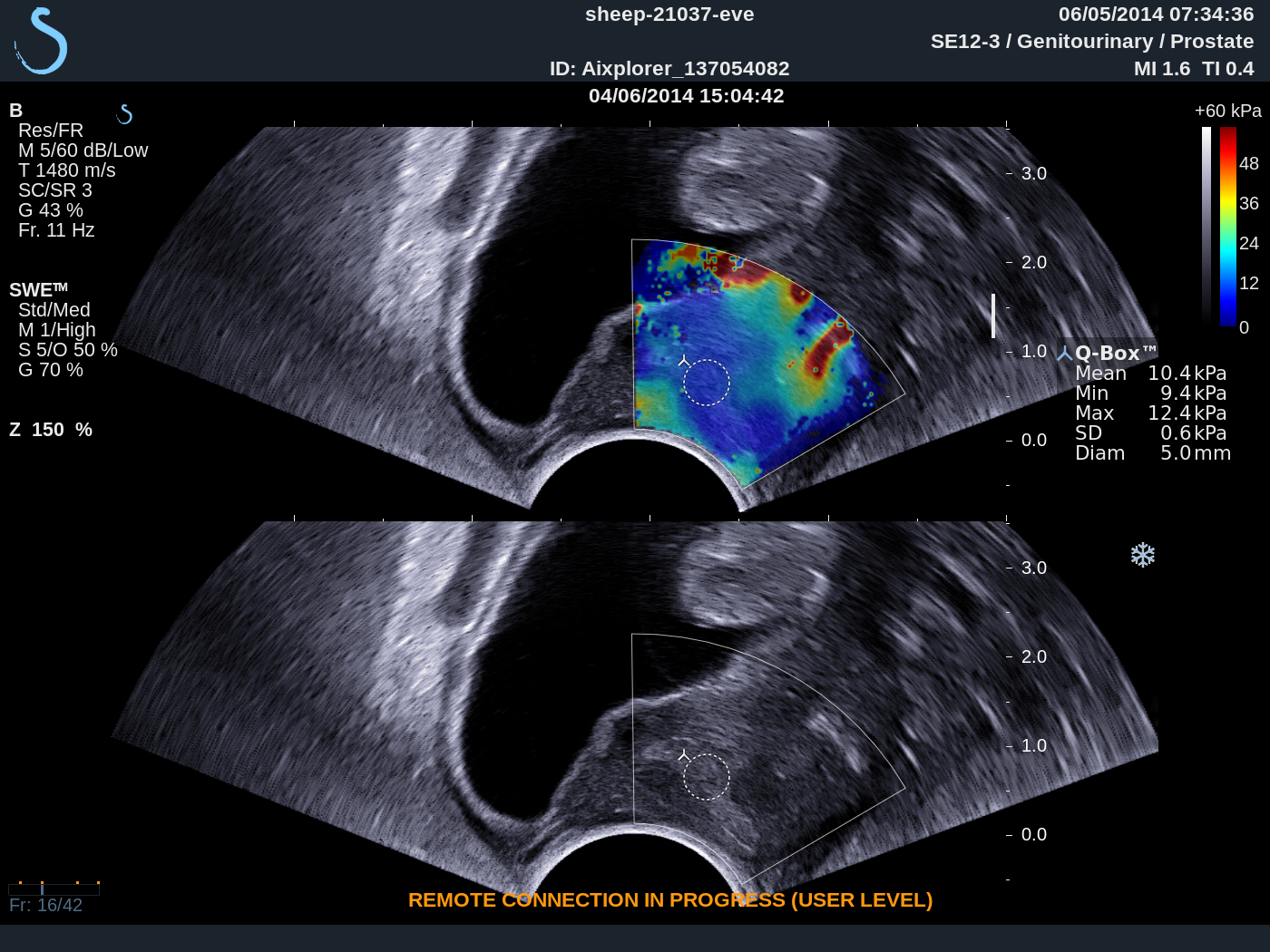

Supplement: S1 Fig — Example of a ROI (white circle) of 5 mm diameter positioned in elastographic image following only anatomical criteria on the conventional B-mode image. (ZIP) [file pone.0133377.s001.zip › S1 Fig/2014_06_04_150442_652.png]

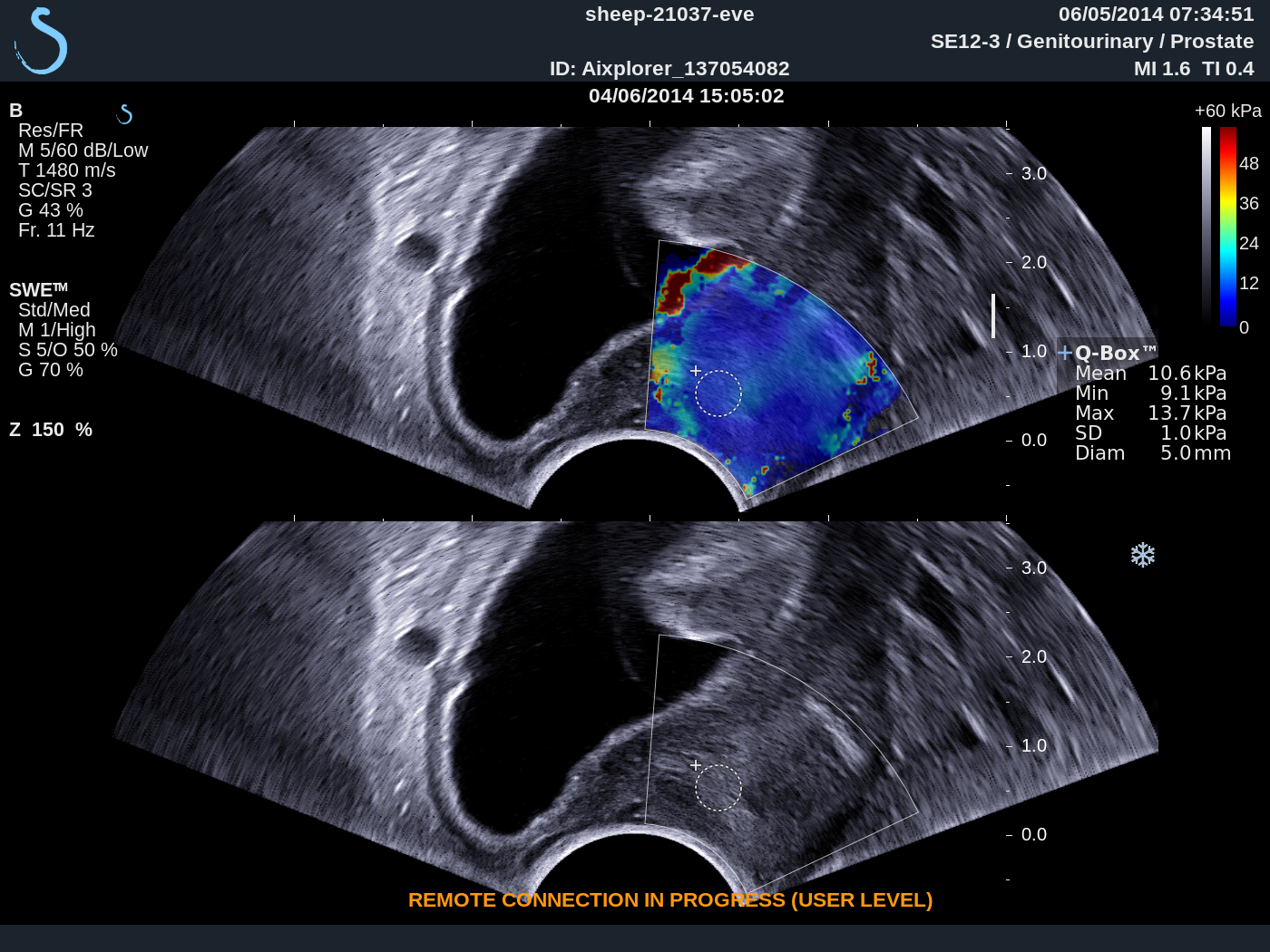

Supplement: S1 Fig — Example of a ROI (white circle) of 5 mm diameter positioned in elastographic image following only anatomical criteria on the conventional B-mode image. (ZIP) [file pone.0133377.s001.zip › S1 Fig/2014_06_04_150502_563.png]

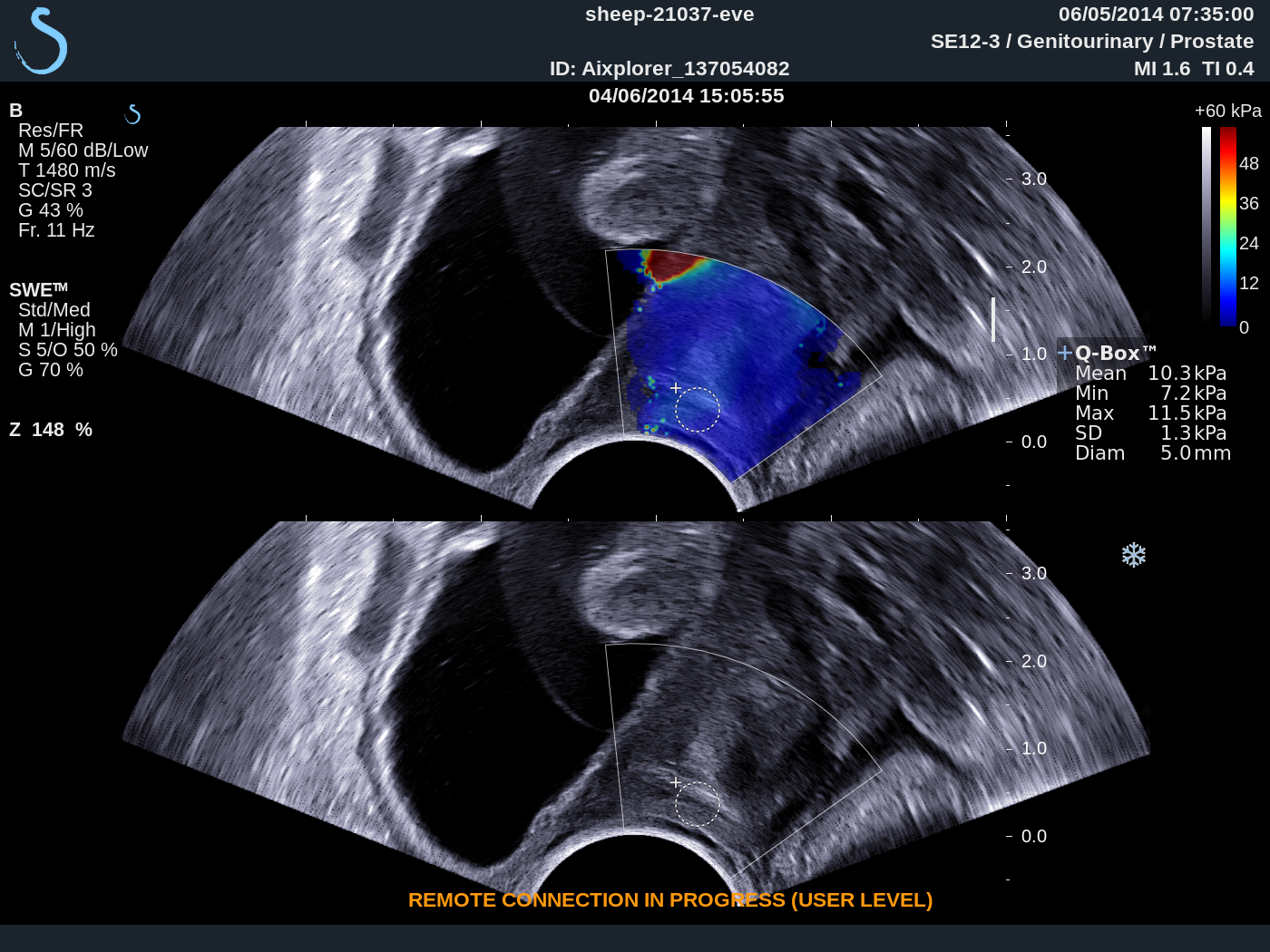

Supplement: S1 Fig — Example of a ROI (white circle) of 5 mm diameter positioned in elastographic image following only anatomical criteria on the conventional B-mode image. (ZIP) [file pone.0133377.s001.zip › S1 Fig/2014_06_04_150555_190.png]
